# Ascending aortic aneurysm repair and surgical ablation for atrial fibrillation

**DOI:** 10.1186/s13019-015-0382-y

**Published:** 2015-11-26

**Authors:** Berhane Worku, Iosif Gulkarov, Charles A. Mack, Leonard N. Girardi, Arash Salemi

**Affiliations:** 1 Department of Cardiothoracic Surgery, Weill Cornell Medical College/New York Presbyterian Hospital, New York, USA; 2 Department of Cardiothoracic Surgery, New York Methodist Hospital, Brooklyn, USA; 3 Department of Cardiothoracic Surgery, New York Hospital Queens, Queens, USA

**Keywords:** Aortic aneurysm, Atrial fibrillation, Ablation

## Abstract

**Background:**

Although surgical ablation of atrial fibrillation is commonly performed during concomitant coronary or valve surgery, it is still only performed in a fraction these cases when indicated, and less often in patients undergoing aneurysm surgery. We describe our experience in patients undergoing ascending aneurysm repair and concomitant atrial fibrillation ablation.

**Methods:**

From January 2004 until November 2011, 40 patients underwent ascending aneurysm repair and atrial fibrillation ablation at our institution and were retrospectively analyzed.

**Results:**

Average age was 67.6 years (43–85). Root replacement was performed in 23 (57.5 %) and arch replacement with circulatory arrest in 18 (45 %). At an average of 41.8 months, 81 % of patients were in sinus rhythm. Operative survival was 100 %, with 1 and 5 year survival of 97.5 and 93.1 %, respectively. Kaplan-Meier analysis revealed improved overall survival in patients with rhythm success (log-rank test *p* = 0.037).

**Conclusions:**

Aortic aneurysm repair with concomitant atrial fibrillation ablation is safe and efficacious despite the requirement for an already extensive procedure with rhythm success rates similar to those quoted in the setting of other procedures. Successful restoration of sinus rhythm improves long term survival and should be considered in patients presenting with aortic aneurysm and atrial fibrillation.

## Background

Atrial fibrillation (AF) is an independent risk factor for stroke and death [1, 2]. It affects up to 1 % of the general population, and 8 % of those over 80 [3]. With the rising age of our society, the incidence of this disease is increasing. Rate control strategies do not address the drop in cardiac output or the risk of thromboembolism associated with loss of atrial contraction, and the benefits associated with rhythm control are negated by the significant side effects of the required medications [4].

The original “cut and sew” Cox-MAZE procedure demonstrated 90–99 % long-term freedom from AF [5, 6]. The development of energy modalities to create similar ablation lesions allowed for the simplification of the procedure. The modified Cox-MAZE III is now commonly performed in patients with AF undergoing concomitant cardiac surgery. Mitral valve repair/replacement (MVR) is most commonly associated with surgical AF ablation as these patients have frequently developed the substrate for AF (i.e. left atrial dilation) and exposure is complimentary [7]. Aortic valve replacement (AVR) and coronary artery bypass grafting (CABG) are also well described in association with surgical AF ablation [7]. Aneurysm surgery is less commonly performed with surgical AF ablation, likely due to the less common association of the two disease states and the perception of an assumed increase in risk of an already extensive procedure. We describe our experience in 40 patients undergoing ascending aneurysm repair and concomitant surgical AF ablation with the goal of demonstrating safety and efficacy.

## Methods

### Patient population

From January 2004 until November 2011, 40 patients underwent ascending aneurysm repair and surgical AF ablation at our institution. Average age was 67.6 years (43–85) and nine (23 %) patients were female. Concomitant procedures were performed as indicated. Rhythm was determined by electrocardiography. We defined the type of AF in accordance with the Heart Rhythm Society/European Heart Rhythm Association/European Cardiac Arrhythmia Society Expert Consensus Statement [8]. Paroxysmal AF is defined as recurrent AF (greater than two episodes) that terminates spontaneously within seven days. Persistent AF is defined as recurrent AF that is sustained for greater than 7 days. Longstanding persistent AF is defined as continuous AF of greater than one year’s duration. Permanent AF is defined as AF whose presence is accepted by the patient (and physician). This study was approved by the Weil Cornell Medical College Institutional Review Board (#1207012764).

### Surgical approach

All cases were performed on cardiopulmonary bypass with cold potassium cardioplegic arrest. Circulatory arrest was utilized as needed for aortic arch reconstructions. Surgical AF ablation is generally performed first after the heart has been arrested, after which mitral repair/replacement is undertaken if indicated. Aortic valve and aneurysm repair/replacement follow. If coronary bypass is indicated, distal anastomoses are performed prior to AF ablation. If circulatory arrest is utilized, AF ablation is generally performed first during the period of systemic cooling.

For the left atrial lesion set, the left atrium is entered via the interatrial groove. Five cryoablation lesions are created for 60 s each at −120° centigrade. The right and left pulmonary veins are isolated separately. A lesion from the right pulmonary vein isolation to the P3 segment of the mitral annulus is created (mitral annulus lesion) as is a connecting lesion from the right to the left pulmonary vein isolation. Finally, a lesion around the base of the left atrial appendage is created which overlaps the left pulmonary vein isolation.

For the right atrial lesion set, the right atrium is opened via a vertical incision and caval lesions to the superior and inferior vena cavae are created. Additional lesions are created from atriotomy to the tricuspid annulus at the anterior/septal commissure and the posterior/septal commissure.

### Demographics, risk factors, and outcomes

Preoperative variables examined included age, gender, type of AF, duration of AF, left ventricular ejection fraction, and left atrial size, as well as comorbidities. Intraoperative variables included lesion sets performed, operative times, and concomitant procedures. Postoperative variables included postoperative complications such as need for pacemaker placement, length of hospital stay, rhythm success, and survival.

### Statistical methods

Continuous variables are expressed as mean +/− standard deviation and categorical variables as frequency and percentage. Continuous variables were compared with the student’s *t*-test and categorical variables were compared with the chi squared test or Fisher’s exact test as appropriate. A p-value of less than 0.05 was considered statistically significant. Kaplan-meier analysis was used to calculate survival rates and the log-rank test was used to determine statistical significance. Multivariable analysis was used to determine independent predictors of rhythm success. All data were analyzed using STATA (StataCorp LP, College Station, TX) or Excel (Microsoft Corporation, Seattle, WA).

## Results

### Baseline characteristics

From January 2004 until November 2011, 40 patients underwent ascending aneurysm repair and surgical AF ablation at our institution. Average age was 67.6 years (43–85) and nine (23 %) patients were female. Preoperative comorbidities are listed in Table 1. Two patients had Marfan syndrome. Twenty (53 %) patients had paroxysmal AF. The average duration of AF was 48.3 months.

**Table 1:** Patient characteristics

	*n* (%)^*^
Age (years)	67.6 (43–85)
Female	9 (22.5)
Diabetes	3 (7.5)
Hypertension	30 (75)
Hyperlipidemia	20 (50)
Chronic renal insufficiency	1 (2.5)
Congestive heart failure	6 (15)
Cerebrovascular accident	5 (12.5)
Reoperative status	2 (5)
Paroxysmal atrial fibrillation	20 (53)
Preoperative left atrial size (centimeters)	4.4 (2.3–8.2)
Preoperative ejection fraction (%)	51.9 (29–80)
Duration of atrial fibrillation (months)	48.3 (1–324)

^*^-Unless otherwise stated

### Intraoperative details

Concomitant procedures are listed in Table 2. Two (5 %) patients underwent reoperation. One had a prior type A dissection repair 12 years prior and presented with aneurysmal dilation of the aortic root and arch with aortic insufficiency, and the other had a prior atrial septal defect closure. One (2.5 %) patient had a prior thoracoabdominal aneurysm repair. Average cardiopulmonary bypass time was 150.8 min and average cardiac ischemic time was 118.4 min. Eighteen (45 %) patients underwent arch or hemiarch reconstructions utilizing circulatory arrest, with an average circulatory arrest time of 21.1 min. All patients underwent a left atrial lesion set, and four (10 %) patients underwent a right atrial lesion set at the surgeons’ discretion.

**Table 2:** Associated procedures

	*n* (%)
Aortic arch replacement:	18 (45)
Hemiarch	13
Total arch	5
Aortic root replacement:	23 (57.5)
Composite valve-graft	18
Valve sparing root replacement	5
Aortic valve procedure without root replacement:	9 (22.5)
Replacement	5
Repair	4
Mitral valve procedure:	9 (22.5)
Replacement	5
Repair	4
Tricuspid valve repair	1 (2.5)
Coronary artery bypass grafting	4 (10)
Atrial septal defect	1 (2.5)

### Postoperative outcomes

Average length of hospital stay was 7.4 days. One patient had a preoperative pacemaker and five (12.8 %) patients required pacemaker placement within the first 30 days for heartblock or sick sinus syndrome. Three (7.5 %) patients required mediastinal reexploration for bleeding and three (7.5 %) required drainage procedures for delayed pericardial effusions (one via pericardiocentesis, one via pericardial window, and one via video assisted thoracoscopic drainage).

Rhythm follow-up at one year or greater was available in 90 % (35/39) of patients. Of these patients, at an average of 41.8 months, 81 % of patients were in sinus rhythm, and 63 % were in sinus rhythm off antiarrhythmic medications. In univariate analysis, predictors of rhythm failure included congestive heart failure (0 vs 33 %; *p* = 0032), longer cardiopulmonary bypass time (146.5 vs 180 min; *p* = 0.006), and longer crossclamp time (112.2 vs 149.3 min; *p* = 0.005 [Table 3]). Longer cardiopulmonary bypass and crossclamp times were similarly predictive of rhythm failure despite use of antiarrhythmic medications. In multivariable logistic regression analysis, no specific factor was predictive of rhythm failure.

**Table 3:** Univariate analysis

	Normal Sinus Rhythm *n* (%)^*^		
	Yes	No	*p*-value
	*n* = 25	*n* = 6	
Female sex	5 (20)	0 (0)	0.553
Age (years)	65.2	67.2	0.669
Diabetes	2 (8)	1 (17)	0.488
Renal insufficiency	0 (0)	1 (17)	0.194
Hypertension	17 (68)	6 (100)	0.298
Hyperlipidemia	11 (44)	3 (50)	1.0
Cerebrovascular disease	3 (12)	2 (33)	0.241
Heart failure	0 (0)	2 (33)	0.032
Root repair	14 (56)	5 (83)	0.363
Arch repair	11 (44)	3 (50)	1.0
Aortic valve procedure	18 (72)	4 (67)	1.0
Mitral valve procedure	4 (16)	1 (17)	1.0
Persistent atrial fibrillation	9 (38)	3 (50)	0.660
Left atrial size (centimeters)	4.1	5.16	0.09
Ejection fraction (%)	52.4	47.7	0.281
Duration (months)	51.5	77	0.606
Bypass time (minutes)	146.5	180	0.006
Ischemia time (minutes)	112.2	149.3	0.005

^*^-Unless otherwise stated

**Fig. 1:**
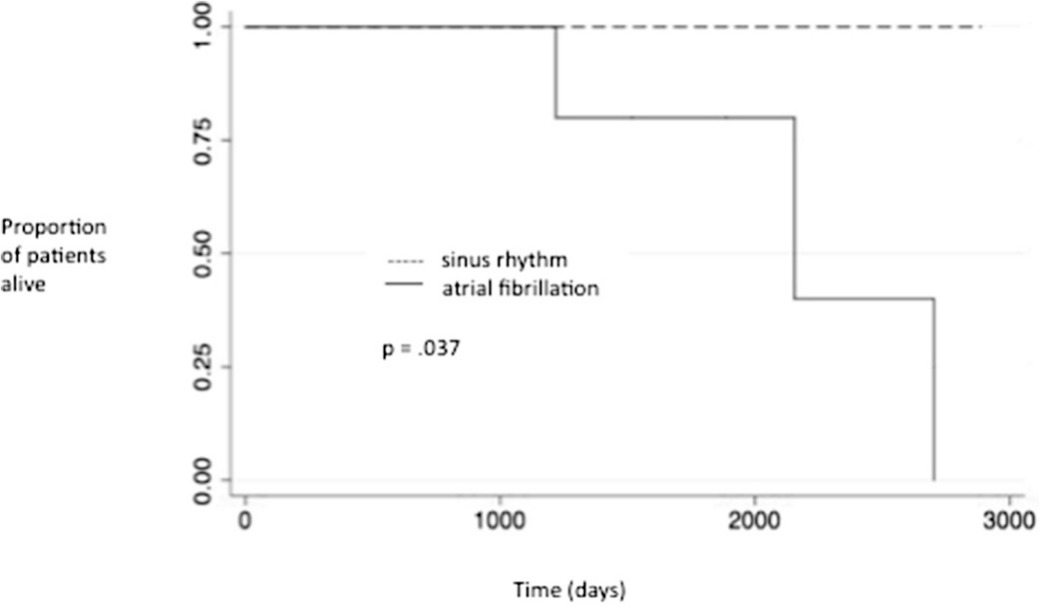
Kaplan-Meier Survival Curves for all patients based on rhythm success

Operative survival (survival to discharge and past 30 days) was 100 %. Overall survival was 90 % at an average of 46 months (16–110 months), with actuarial one and five year survivals of 97.5 and 93.1 %, respectively. Kaplan-Meier analysis revealed improved overall survival in patients with rhythm success (*p* = 0.037 [Fig. 1]).

## Discussion

Although the initial Cox-MAZE procedures demonstrated high rhythm success rates, their invasiveness precluded widespread application. With the development of energy sources that can create the required lesion sets with greater ease and minimal morbidity, surgical ablation had become more frequently utilized, especially in the setting of concomitant MVR [7]. The development of novel techniques and minimally invasive approaches has resulted in the application of surgical AF ablations during off-pump procedures [9] or as a standalone procedure [10]. However, up to 60 % of patients with AF undergoing cardiac surgery do not undergo AF ablation [7]. This is unfortunate as patients with preoperative AF undergoing cardiac surgery have a worse prognosis than those without AF [11–15], and AF ablation reverses this [14, 15].

Preoperative AF has been shown to correlate with operative mortality after CABG [12], AVR/CABG, and AVR/MVR [13] and midterm and longterm mortality after AVR, AVR/CABG, AVR/MVR [11, 13], and MVR [14]. As expected, patients with preoperative AF presented with elevated comorbidity rates, but these relationships persisted despite controlling for confounding factors [11–14]. In a study of 281,567 patients from the Society of Thoracic Surgeons National Adult Cardiac Surgery Database undergoing CABG by Ad et. al., preoperative AF was associated with increased rates of reoperation, respiratory failure, renal failure, stroke, increased length of stay, and mortality despite adjustment for confounding factors. It was suggested that AF be considered a risk factor in risk-prediction models, as was recently done by the Society of Thoracic Surgeons risk score [12].

Just as it has been well documented that preoperative AF is a risk factor for poorer outcomes after cardiac surgery, studies have demonstrated that this can be reversed by successful AF ablation. Preoperative AF is an independent predictor of longterm mortality after MVR [14]. Similarly, preoperative AF is associated with lower freedom from thromboembolic complications [14] and surgical AF ablation is protective [14, 16], lowering thromboembolic event rates to those seen in patients without preoperative AF [14]. These reductions in thromboembolic events after AF ablation are seen despite continued anticoagulation for mechanical valves [14, 16]. In a propensity matched analysis of 3262 patients with AF and 2449 patients without AF undergoing cardiac surgery, patients with preoperative AF had reduced survival compared to patients with no AF. AF ablation reversed this relationship, improving survival rates to those seen in patients without AF. Similarly, successfully ablated patients had better survival than unsuccessfully ablated patients [15]. In addition to thromboembolism and mortality rates, preoperative AF has been shown to correlate with milder improvements in ejection fraction and greater tricuspid regurgitation after MVR [14], and surgical AF ablation results in greater improvement in ejection fraction [14, 16] and tricuspid regurgitation [16] postoperatively.

Despite several such documented benefits of concomitant surgical AF ablation during cardiac surgery, its less than routine application in patients with preoperative AF requires investigation. Amongst the potential explanations are risks related to additional crossclamp and bypass time, additional maneuvers for exposure, and increased rates of postoperative bradyarrhythmias requiring pacemaker implantation. Concomitant AF ablation results in longer crossclamp (9 to 37 min longer) and cardiopulmonary bypass (9 to 42 min longer) times [7, 16] and increased pacemaker requirements [7, 17], but even after controlling for confounders, surgical ablation has been shown to be safe with no increased risk of operative mortality or major morbidity [7]. Surgical ablation has been routinely performed concomitant to valve/CABG [7, 11, 13, 15], multiple valves [13, 14, 16], and reoperative [18] procedures with good outcomes. However, no reports of aortic aneurysm surgery with surgical ablation exist.

In the current study, aneurysm repair with concomitant AF ablation was performed with 100 % operative survival and greater than 90 % long term survival, with no complications attributable to the ablation procedure. Surgical ablation was performed in the setting of extensive procedures with already prolonged cardiopulmonary bypass and crossclamp times involving additional valve, coronary, or arch surgery with circulatory arrest in a high proportion of patients. Furthermore, survival was improved in patients with successful ablation. This is the first study to our knowledge to demonstrate the safety and efficacy of surgical AF ablation in patients undergoing aortic aneurysm surgery. Prolonged cardiopulmonary bypass time has been associated with increased blood transfusion requirements, intensive care unit length of stay [19], and mortality [20] after ascending aortic and aortic arch repair. While we did not compare operative times to a control group of patients undergoing aortic aneurysm surgery without surgical AF ablation, all ablations were performed via a left +/− right atriotomy which in the majority of cases was not otherwise required for exposure for the given procedure. Thus it is likely that operative times were significantly prolonged for the ablation. Finally, rhythm success rates were similar to those published elsewhere despite a high percentage of patients with more persistent forms of AF.

Limitations of this study include those inherent to a retrospective analysis using chart review and include incomplete data, potential inaccuracies in data, and potential for selection bias. In addition, because of differences in clinical practice across centers, extrapolation of results may be of limited value. Finally, the small sample size, heterogeneity of surgical procedures, and lack of a control group limit usefulness of statistical analyses.

## Conclusions

In conclusion, aortic aneurysm repair with concomitant surgical AF ablation is safe and efficacious despite the requirement for an already extensive procedure. Excellent short and long term survival can be achieved as can rhythm success rates similar to those quoted in the setting of other procedures. Successful restoration of sinus rhythm improves long term survival and should be considered in patients presenting with aortic aneurysm and AF.

## Abbreviations

AF: Atrial fibrillation
MVR: Mitral valve repair/replacement
AVR: Aortic valve replacement
CABG: Coronary artery bypass grafting
